# Both clinical and environmental *Caulobacter* species are virulent in the *Galleria mellonella* infection model

**DOI:** 10.1371/journal.pone.0230006

**Published:** 2020-03-12

**Authors:** Gabriel M. Moore, Zemer Gitai

**Affiliations:** Department of Molecular Biology, Princeton University, Princeton, NJ, United States of America; Centre National de la Recherche Scientifique, Aix-Marseille Université, FRANCE

## Abstract

The *Caulobacter* genus, including the widely-studied model organism *Caulobacter crescentus*, has been thought to be non-pathogenic and thus proposed as a bioengineering vector for various environmental remediation and medical purposes. However, *Caulobacter* species have been implicated as the causative agents of several hospital-acquired infections, raising the question of whether these clinical isolates represent an emerging pathogenic species or whether Caulobacters on whole possess previously-unappreciated virulence capability. Given the proposed environmental and medical applications for *C*. *crescentus*, understanding the potential pathogenicity of this bacterium is crucial. Consequently, we sequenced a clinical *Caulobacter* isolate to determine if it has acquired novel virulence determinants. We found that the clinical isolate represents a new species, *Caulobacter mirare* that, unlike *C*. *crescentus*, grows well in standard clinical culture conditions. *C*. *mirare* phylogenetically resembles both *C*. *crescentus* and the related *C*. *segnis*, which was also thought to be non-pathogenic. The similarity to other Caulobacters and lack of obvious pathogenesis markers suggested that *C*. *mirare* is not unique amongst Caulobacters and that consequently other Caulobacters may also have the potential to be virulent. We tested this hypothesis by characterizing the ability of Caulobacters to infect the model animal host *Galleria mellonella*. In this context, two different lab strains of *C*. *crescentus* proved to be as pathogenic as *C*. *mirare*, while lab strains of *E*. *coli* were non-pathogenic. Further characterization showed that *Caulobacter* pathogenesis in the *Galleria* model is mediated by lipopolysaccharide (LPS), and that differences in LPS chemical composition across species could explain their differential toxicity. Taken together, our findings suggest that many *Caulobacter* species can be virulent in specific contexts and highlight the importance of broadening our methods for identifying and characterizing potential pathogens.

## Author summary

Bacterial species have historically been classified as either capable of causing disease in an animal (pathogenic) or not. *Caulobacter* species represent a class of bacteria that were thought to be non-pathogenic. Caulobacters have been widely studied and proposed to be used for various industrial and medical applications due to their presumed safety. However, recent reports of human *Caulobacter* infections raised the question of whether disease-causing Caulobacters have acquired special factors that help them cause disease or whether the ability to infect is a more general feature of most Caulobacters. By combining genomic sequencing and animal infection studies we show that a clinical *Caulobacter* strain is similar to lab Caulobacters and that all Caulobacters studied can cause disease in a model host. We explore the mechanism of this infectivity and show that it is due to the production of a toxic factor that is made by all *Caulobacter* cells. We also provide a possible explanation for why Caulobacters have not traditionally been isolated from human patients, owing to their inability to tolerate the salt levels used in most medical culturing systems.

## Introduction

The free-living, gram-negative genus *Caulobacter* was first described and classified as a group of rod-shaped, stalk possessing bacteria in 1935 [1, 2]. Since their identification, *Caulobacter* have been observed in rhizosphere, soil, and aqueous environments, including drinking water reservoirs [3, 4]. Historically, this genus has been considered non-pathogenic due to lack of presence in infection cases, no obvious pathogenicity islands, and increased bacterial mortality at human body temperatures [5]. However, the last two decades have seen several reports of symptomatic infections associated with *Caulobacter* species [6–10]. All reported cases of *Caulobacter* infections appear to be hospital-acquired by immunocompromised patients, suggesting that these infections are opportunistic. None of the *Caulobacter* isolates associated with human infection have been previously sequenced. Consequently, it remains unclear whether clinical isolates have acquired virulence mechanisms absent from other Caulobacters, or if *Caulobacter* species generally have the capacity for human disease in the right context.

Among *Caulobacter* species, *Caulobacter crescentus* is the best characterized and most widely studied in laboratory settings [11]. *C*. *crescentus* has been primarily used as a model organism for understanding bacterial cell-cycle progression due to its highly regulated asymmetrical division and dimorphic lifestyle [12, 13]. Because of its available molecular tools, ability to display proteins in its surface layer (S-layer), and assumed non-toxicity to humans, *C*. *crescentus* has been proposed to be a powerful vector for a wide range of bioengineering applications [14, 15]. For example, *C*. *crescentus* has been engineered as a biosensor for uranium [16], a bioremediation tool for heavy metals [17], an anti-tumor immunization technique [18], and an anti-viral microbicide in humans [19, 20]. Thus, understanding the potential pathogenicity of this bacterium is crucial before its industrial use.

Here we obtained and sequenced a *Caulobacter* isolate from a reported human infection [7] to determine if it contains conspicuous virulence determinants or is similar to previously-characterized Caulobacters. We found that the clinical isolate represents a new species with similarities to both *C*. *crescentus* and another environmental species *Caulobacter segnis*. The lack of pathogenicity islands and similarity to lab strains of *C*. *crescentus* suggested that the potential of this clinical isolate to be an opportunistic pathogen may be a general feature of Caulobacters. We confirmed this hypothesis by turning to the *Galleria mellonella* model animal host. The clinical *Caulobacter* isolate and lab strains of *C*. *crescentus* exhibited similar virulence, which were both significantly higher than non-pathogenic lab strains of *E*. *coli* or other members of the alphaproteobacteria class. Further characterization revealed that *Caulobacter* virulence in the *G*. *mellonella* model is mediated by toxicity induced by lipopolysaccharide (LPS), whose composition was previously shown to differ between Caulobacters and *E*. *coli*. Thus, our findings establish that *Caulobacter* species can act be virulent when able to sabotage their hosts in the right context.

## Results

### Clinical *Caulobacter sp*. *SSI4214* shares homology with soil- and freshwater-associated species of *Caulobacter*

**Fig 1:**
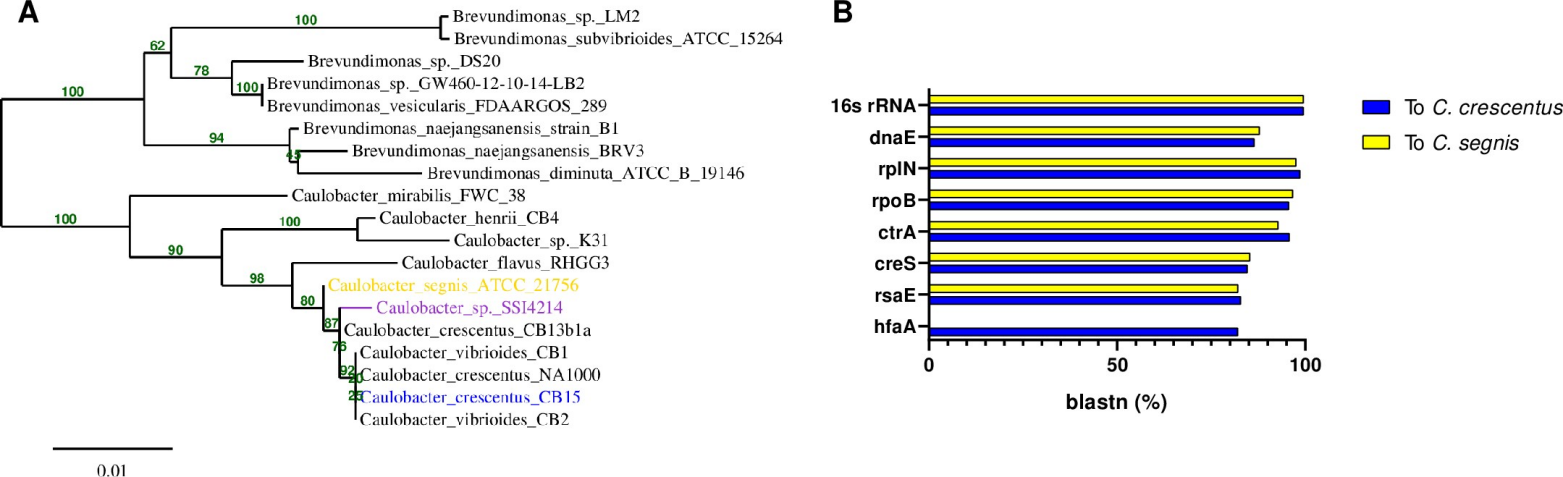
Genomic comparison of *Caulobacter mirare* SSI4214 to related *Caulobacter* species. (A) Phylogenetic tree containing SSI4214 along with closely related species. Numbers indicate bootstrapping confidence values for nodes after 100 replicates. Bar represents average nucleotide substitution/site (B) BLAST values of conserved and *Caulobacter* genus-specific genes.

To genomically characterize a clinical *Cauloacter* isolate, we obtained a clinical strain of *Caulobacter* species isolated from the dialysis fluid of a 64-year-old man in Denmark with peritonitis [7]. There was only one bacterial species that could be cultured from the peritoneal fluid using Danish blood agar medium, and the infection responded to gentamycin treatment suggesting that this species was the likely cause of the infection [7]. Imaging of the cultured bacteria revealed a crescent-shaped morphology similar to that of *Caulobacter crescentus* and 16S ribosomal profiling showed 99.5% homology between the clinical isolate (*Caulobacter sp*. SSI4214) to a common laboratory *C*. *crescentus* strain CB15 [7]. We performed next-generation Illumina sequencing on the *Caulobacter sp*. SSI4214 strain and created a draft genome assembly to understand the isolate’s relationship to other *Caulobacter* species. Analysis of the 16S rRNA gene obtained from Illumina sequencing confirmed the initial report, with 99.5% similarity to *C*. *crescentus*. However, phylogenetic reconstruction comparing the 16S sequences of all available whole-genome *Caulobacter* species revealed that *Caulobacter sp*. SSI4214 resides in its own separate clade within the *Caulobacter* genus, between *Caulobacter crescentus* and *Caulobacter segnis* (Fig 1A). SSI4214 was also similar to both *C*. *crescentus* and *C*. *segnis* with respect to overall GC content and two-way average nucleotide identity (Table 1).

**Table 1:** Genomic features of *Caulobacter mirare* draft genome assembly compared to *C*. *crescentus* and *C*. *segnis*.

	*C*. *crescentus (complete)*	*C*. *mirare (draft genome)*	*C*. *segnis (complete)*
Genome Size (bps	4,016,947	4,789,750	4,655,622
GC Content (%)	67.21	67.51	67.67
Predicted-Coding Genes	3,819	4,329	4,330
Pathogenicity Islands	0	0	0
Average Nucleotide Identity (%) of SSI4214 to	83.88		84.75

Annotation of the SSI4214 genome allowed us to compare homology of its genes to those of *C*. *crescentus* and *C*. *segnis*, including both broadly-conserved and *Caulobacter-*specific genes [21]. Overall, SSI4214 is predicted to encode 4,329 protein-encoding genes. This number is similar to that of the *C*. *segnis* genome (4,330 genes), and larger than *C*. *crescentus* (3,819) (Table 1) [22, 23]. Among broadly-conserved genes, subunits of DNA polymerase, RNA polymerase, and ribosomes all exhibited at least 86% sequence similarity to both *C*. *crescentus* and *C*. *segnis*. SSI4214 also possesses clear homologs of many *Caulobacter-*specific genes including the cell-cycle regulator *ctrA*, the curvature determinant *creS*, the S-layer secretion protein *rseE*, and the holdfast attachment protein *hfaA* (Fig 1B). We note that *C*. *segnis* does not possess a majority of the holdfast synthesis genes, including *hfaA* (Fig 1B) [22].

Bacterial species are functionally defined as genomes with at least 95% average nucleotide identity [24]. To define the species to which SSI4214 belongs we thus performed an average nucleotide identify analysis with its two closest relatives, *C*. *crescentus* and *C*. *segnis*. SSI4214 exhibited only 83% identity to *C*. *crescentus* and 85% identity to *C*. *segnis* (Table 1), indicating that SSI4214 represents a distinct species in the *Caulobacter* genus. Following the convention of the International Code of Nomenclature of Prokaryotes [25], we named this new species *Caulobacter mirare*, as *mirare* is the Latin root for *mirage* (by way of the French *se mirer*) (Table 1, S1 Fig). The observations that *C*. *mirare* is more similar to *C*. *segnis* with respect to gene number but more similar to *C*. *crescentus* with respect to holdfast gene content supports its placement as an independent clade in between the two related species. Importantly, like *C*. *crescentus* and *C*. *segnis*, no known annotated virulence factor homologues or pathogen-associated genes are predicted to be present in *C*. *mirare* [26]. Thus, genome sequencing suggests that the pathogenicity of *C*. *mirare* is not the result of acquisition of a significant pathogenicity island, and that this clinical isolate broadly resembles environmental *Caulobacter* isolates that were previously considered non-pathogenic. In other words, the seemingly higher pathogenicity of *Caulobacter mirare* could simply be a “mirage.”

### *Caulobacter mirare* identification in infection made possible due to difference in culturability

**Fig 2:**
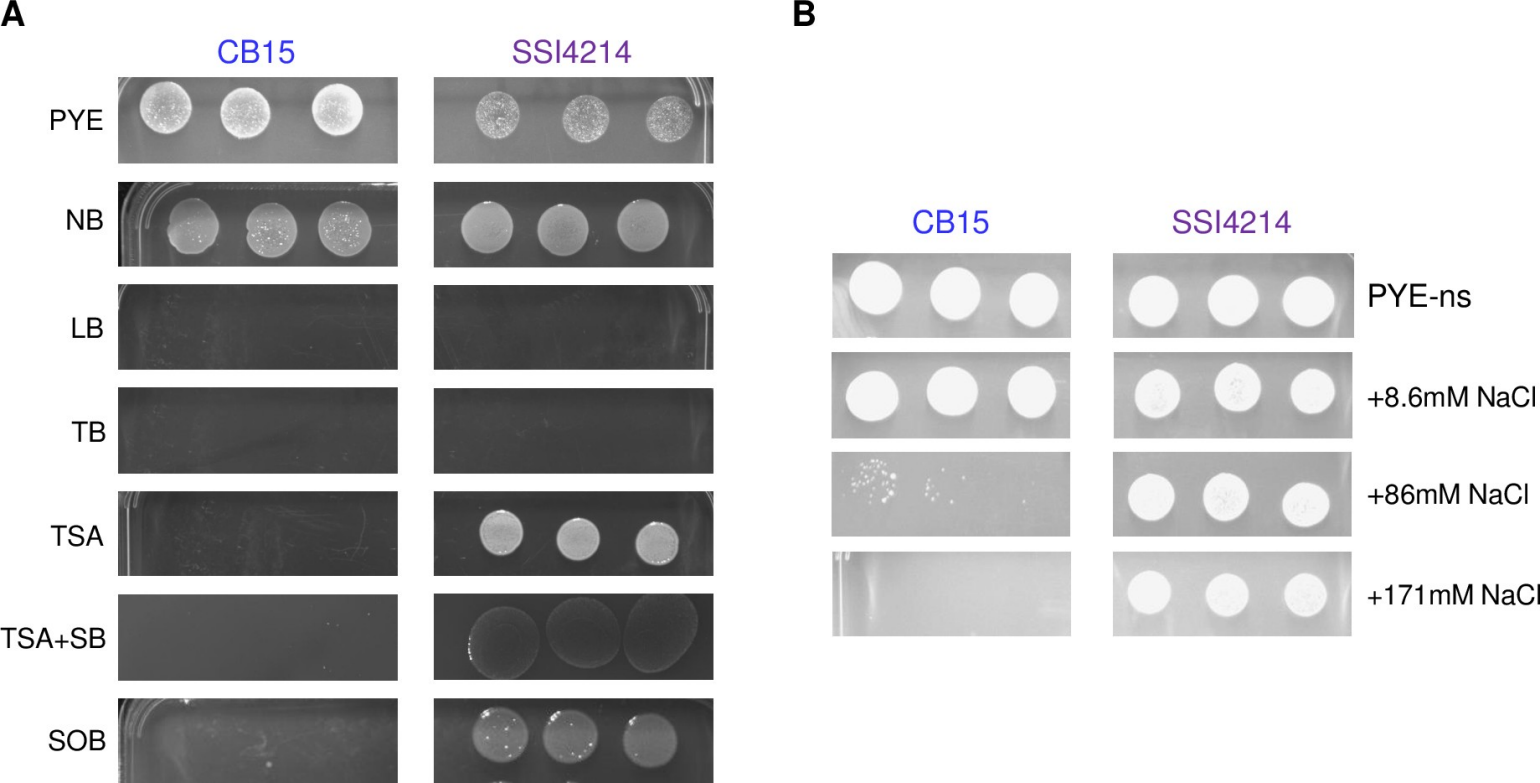
Culturability of *Caulobacter crescentus* (CB15) and *Caulobacter mirare* (SSI4214). (A-B) Three replicates each of 10^−3^-diluted overnight culture of CB15 (left) or SSI4214 (right) on various media. (A) PYE = peptone-yeast extract, NB = nutrient broth, LB = luria broth, TB = terrific broth, TSA = tryptic soy agar, TSA+SB = tryptic soy agar + 5% sheep blood, SOB = super optimal broth. (B) PYE-ns = PYE without added salts, +8.6mM NaCl = PYE-ns with addition of 8.6 mM NaCl, +86mM NaCl = PYE-ns with addition of 86 mM NaCl, +171 mM NaCl = PYE-ns with addition of 171 mM NaCl.

Caulobacters are ubiquitously present in water systems and genomic analysis suggests that *Caulobacter mirare* may not possess novel mechanisms that grant greater infection potential than environmental isolates. This raises the question of why Caulobacters have not been more commonly associated with human infections. One possibility is that *Caulobacter* infections are more common than typically appreciated but that Caulobacters are not readily isolated by culturing-based clinical identification methods. Thus, we compared the culturing requirements of *C*. *crescentus* and *C*. *mirare*. *C*. *mirare* was isolated using Danish blood agar plates [7], and we confirmed that the SSI4214 strain indeed grows on sheep’s blood agar (Fig 2A). In contrast, CB15 *C*. *crescentus* was unable to grow on sheep’s blood agar (Fig 2A). To determine the root cause of this difference, we compared *C*. *crescentus* and *C*. *mirare* growth on several complex media. Both species grew robustly on peptone-yeast extract agar, the standard culturing medium for CB15, and nutrient agar. Media with higher salt concentrations, such as Luria broth and terrific broth, did not allow for growth of either *Caulobacter* species. Meanwhile, lower salt-containing media such as tryptic soy agar and super optimal broth, promoted the growth of *C*. *mirare* but not *C*. *crescentus* (Fig 2A).

To directly determine if salt content is the relevant growth-determining difference in these media we plated both *Caulobacter* species on PYE in which we replaced the normal MgSO_4_ salt with varying amounts of NaCl. Both species still grew on modified PYE with no salt added (Fig 2B). We then increased the NaCl content of the modified PYE and found that while both *Caulobacter* species grew well at 8.6 mM NaCl, *C*. *mirare* continued to grow well at 86 mM and 171 mM NaCl, while *C*. *crescentus* grew poorly at 86 mM NaCl and failed to grow at all at 171 mM NaCl (Fig 2B). These data suggest that the increased salt tolerance of *C*. *mirare* may explain why this strain could be cultured from an infected patient.

### Both *C*. *mirare* and *C*. *crescentus* decrease healthspan in *Galleria mellonella*

Given the genomic similarity between *C*. *mirare* and *C*. *crescentus* we sought to directly compare their pathogenic potential in an *in vivo* host model. *Galleria mellonella*, the greater wax moth, has emerged as a useful system for assessing infection potential due to its relatively short lifespan and ability to inject a defined inoculum of bacteria [27]. Additionally, *Galleria* produces melanin upon infection as part of its immune response, providing a robust visual readout for host health. The process of melanization is irreversible such that even if *Galleria* successfully eliminates the cause of infection, it maintains a dark coloration that corresponds to the degree of its immune response [28, 29].

**Fig 3:**
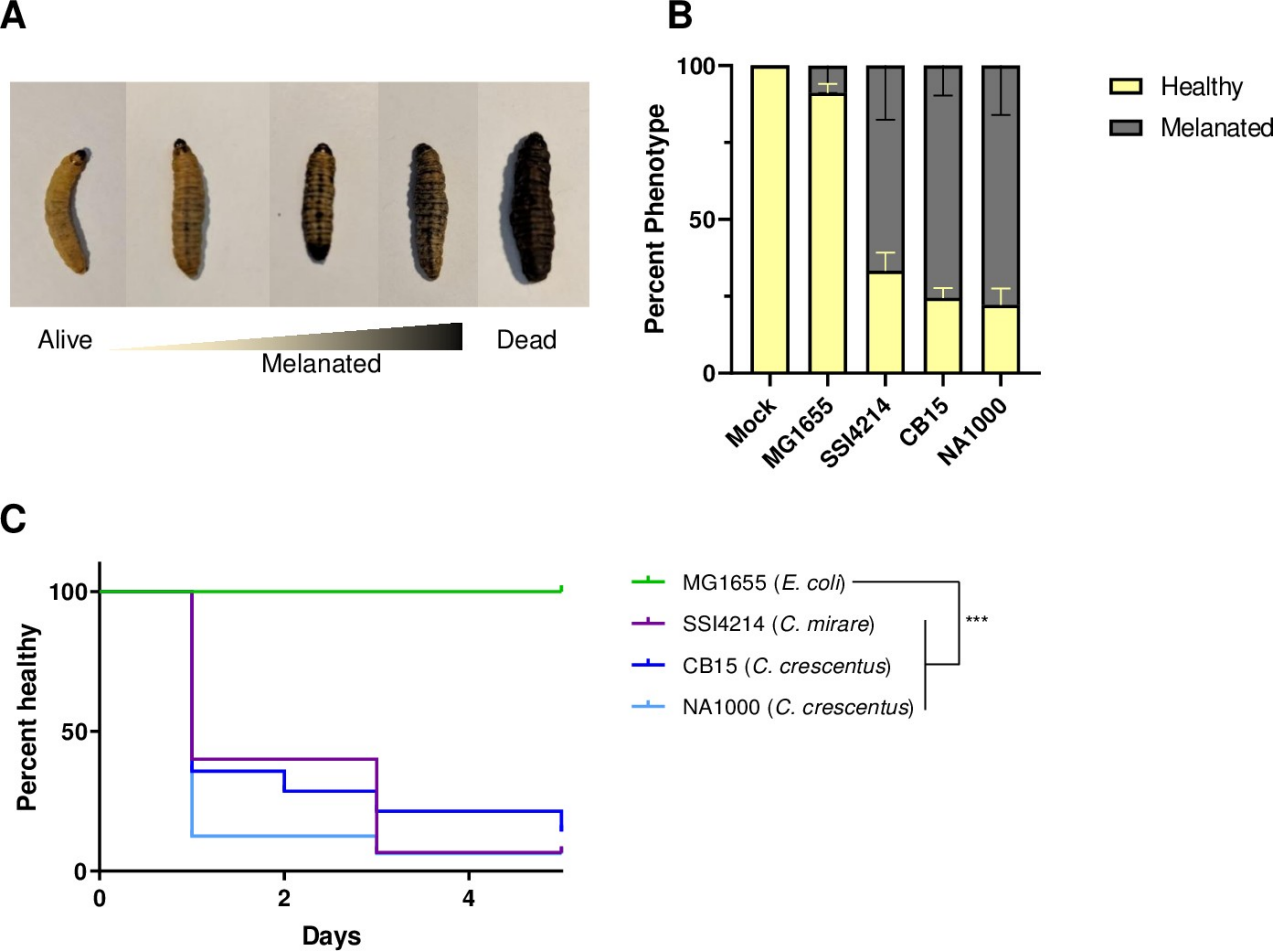
*Galleria mellonella* healthspan decreases upon *Caulobacter* infection. (A) Example images of phenotypes considered for scoring in healthspan assay. (B) Percentage of worms scored as healthy or melanated 24 hours post-inoculation. Error bars represent standard error for three biological replicates. (C) Kaplan-Meier survival analysis for *C*. *crescentu*s strains CB15 and NA1000, *E*. *coli* strain MG1655 and *C*. *mirare* strain SSI4214. Survival curve shown is one representative cohort (n = 15) of three biological replicates (Mantel-Cox test for statistics, ***P < .001).

To quantitatively assay bacterial virulence, we injected *Galleria* with similar numbers of exponentially growing bacteria and monitored melanization after 24 hours, which included fatal events (Fig 3A). As a negative control, we confirmed that mock injections of *Galleria* with water had no effect on melanization. Injection with a lab *E*. *coli* MG1655 strain also had no effect on *Galleria* melanization, indicating that not all bacteria are pathogenic towards *Galleria* (Fig 2B) [30]. In contrast, injection with *C*. *mirare* resulted in significant melanization within 24 hours (Fig 3B), suggesting that *Galleria* could be a useful model for studying *C*. *mirare* pathogenesis. Interestingly, injection with two different lab strains of *C*. *crescentus*, CB15 and NA1000, also resulted in significant *Galleria* melanization within 24 hours (Fig 3A) [31]. The extent of the pathogenesis of the lab *C*. *crescentus* strains towards *Galleria* was comparable to that of the clinical *C*. *mirare* strain.

To follow the dynamics of virulence we performed a healthspan assay by monitoring melanization as a function of time after injecting *E*. *coli* (MG1655), *C*. *mirare* (SSI4214), and *C*. *crescentus* (CB15 and NA1000). Even five days post injection, no melanization was observed with *E*. *coli*, validating its use as a non-pathogenic control (Fig 3C). Meanwhile, significant melanization was observed within 1 day of injecting any of the *Caulobacter* strains and increased as a function of time (Fig 3C). *Galleria* injected with the clinical *C*. *mirare* and lab *C*. *crescentus* strains displayed similar healthspans (Fig 3C). Together these data suggest that *C*. *mirare* can be virulent towards at least some animal hosts, consistent with its clinical isolation and pathology. However, *C*. *mirare* virulence is not unique, but rather a feature it shares with environmental isolates of *C*. *crescentus*.

### Melanization is induced by a dose-dependent, cell-associated factor that does not require live cells

**Fig 4:**
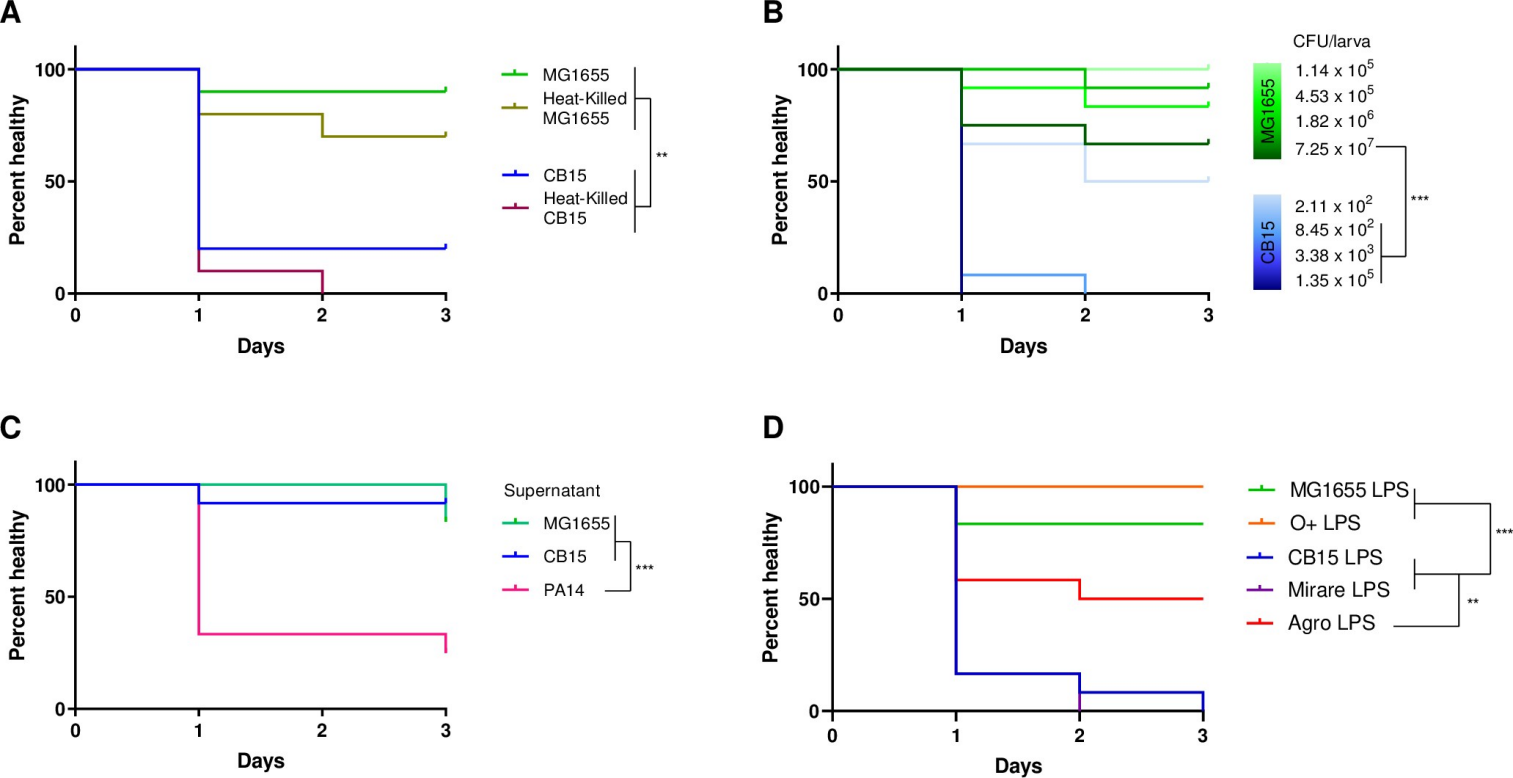
Healthspan decreases in response to cell-associated factor LPS in a dose-dependent manner. (A) Healthspan of *Galleria* upon injection of 5 μL of OD_660_ ~ 0.5 (exponentially-growing) live and heat-killed CB15 and MG1655 (B). Healthspan of *Galleria* upon injection with serial dilutions of overnight CB15 or MG1655. (C) Healthspan of *Galleria* upon injection of supernatant derived from overnight cultures of CB15, MG1655, or *Pseudomonas aeruginosa* strain PA14. (D) Healthspan of *Galleria* upon injection of purified LPS from CB15, *C*. *mirare*, MG1655, O-antigen possessing *wbbL+ E*. *coli* MC4100, and *A*. *tumefaciens* A136. All survival curves are a representative cohort (n = 10–15) of three biological replicates (Mantel-Cox test for statistics, **P < 0.01, ***P < .001).

Since *C*. *crescentus* infected *Galleria* as well as *C*. *mirare* but is more experimentally tractable, we focused our efforts on characterizing the mechanism of *Caulobacter* pathogenesis on *C*. *crescentus*. We first determined whether *Galleria* melanization requires *C*. *crescentus* growth within the host by heat-killing exponentially-growing bacterial cells prior to injection. Using the same starting number of bacterial cells, heat-killed *C*. *crescentus* induced similar melanization to living cells (Fig 4A). Thus, the melanization of *Galleria* by *C*. *crescentus* is not merely a secondary consequence of bacterial growth within the host or outcompeting the host for nutrients. We consequently hypothesized that symptomatic infection is induced via a toxic or immune stimulating factor. A hallmark of toxin-associated pathogenesis is quantitative dependence on bacterial load. To assess the bacterial load required to cause an infection phenotype, we injected *Galleria* with four-fold serial dilutions of overnight cultures of CB15 and MG1655 and performed healthspan assays. For both *C*. *crescentus* and *E*. *coli*, we observed the expected dose-dependence of infection, with increased melanization as a function of increased numbers of bacteria injected (Fig 4B). This experiment also reinforced the difference in pathogenic potential of the two bacterial species, as the lowest number of *C*. *crescentus* injected, (~10^3^), caused more melanization than even the highest number of *E*. *coli* injected (~10^7^).

To determine whether the cytotoxicity of *C*. *crescentus* is due to a secreted or cell-associated factor, we injected *Galleria* with *C*. *crescentus-*conditioned media. Specifically, we centrifuged an overnight *C*. *crescentus* culture capable of inducing *Galleria* melanization at low speeds (5700 g) to remove bacterial cells and cell-associated factors and injected the supernatant that retains secreted factors. CB15 and MG1655 conditioned media did not induce *Galleria* melanization (Fig 4C). As a positive control to confirm that it is possible to induce melanization with secreted toxins we also isolated conditioned media from *Pseudomonas aeruginosa* strain PA14, which is known to secrete exotoxins (Fig 4C) [32]. We confirmed that PA14-conditioned media induced *Galleria* melanization, suggesting that the *C*. *crescentus* toxic factor is not secreted.

### *G*. *mellonella* pathogenesis by *Caulobacter* is mediated by LPS

We next sought to identify specific factors associated with *C*. *crescentus* that could be impacting *Galleria* health and viability. The outermost surface layer of *C*. *crescentus* is the paracrystalline S-layer, which is made up of a polymer of the S-layer protein, RsaA [14]. Deletion of the S-layer protein (Δ*rsaA*) results in a similar healthspan profile to wild-type *C*. *crescentus*, indicating that RsaA is not required for toxicity (S2 Fig). The S-layer is anchored to the membrane via lipopolysaccharides (LPS), which have been demonstrated in other bacterial species to act as an immunostimulant in *Galleria* through upregulation of prophenoloxidase (PPO) [33, 34]. We isolated LPS from *C*. *crescentus* and injected it into *Galleria* at concentrations similar to those found in the number of bacteria sufficient to induce melanization. Interestingly, *C*. *crescentus* LPS alone was able to induce melanization. Furthermore, *C*. *mirare* LPS induced melanization to the same extent as that of *C*. *crescentus* (Fig 4D). Meanwhile, LPS purified from non-pathogenic *E*. *coli* MG1655 was unable to induce melanization (Fig 4D). These results suggest that LPS is sufficient to explain the toxicity of *C*. *crescentus* and *C*. *mirare* and that differences between *Caulobacter* and *E*. *coli* MG1655 LPS might explain their difference in virulence towards *Galleria*.

There are several known differences between the LPS of *E*. *coli* MG1655. For example, *Caulobacter* LPS is linked to O-antigen, and O-antigen is not present in MG1655 due to several well-characterized mutations [35]. To see if the O-antigen causes the melanization response, we obtained a derivative of MG1655 whose O-antigen production has been restored [36]. We observed that LPS purified from these bacteria failed to induce melanization when injected into Galleria, suggesting that the O-antigen is not sufficient to explain the toxicity of *Caulobacter* LPS (Fig 4D). *C*. *crescentus* and *C*. *mirare* are alpha-proteobacteria while *E*. *coli* belong to different bacterial class, gamma-proteobacteria. These classes of bacteria are known to possess different LPS composition, especially in terms of Lipid A backbone [37]. To see if the difference in LPS toxicity extends to more distantly related alpha-proteobacteria, we purified LPS from *Agrobacterium tumefaciens* [38]. *A*. *tumefaciens* is predominantly a plant pathogen but has also been associated with some human infection cases when patients are immunocompromised [39, 40]. LPS extracted from *A*. *tumefaciens* was capable of melanizing *Galleria*, but to a lesser extent than LPS from *Caulobacter* species. These results suggest that species-specific differences in the chemical composition of LPS could be sufficient to explain the differences in their ability to melanize *Galleria* (Fig 4D).

## Discussion

Our work demonstrates that both the clinical *C*. *mirare* and environmental *Caulobacter* species can be virulent towards *Galleria* with similar degrees of toxicity. Not all bacteria can perturb *Galleria* healthspan, as lab strains of *E*. *coli* proved non-pathogenic in this context (Fig 3). Furthermore, sequencing and analysis of the *C*. *mirare* genome indicated that this clinical isolate is similar to *C*. *crescentus* and related Caulobacters that were also considered to be non-pathogenic like *C*. *segnis* (Fig 1). *C*. *mirare* does not appear to have acquired any clear pathogenicity islands or virulence factors [26]. Coupled with its similar extent of virulence as *C*. *crescentus*, our findings thus suggest that *C*. *mirare* is not unique in its ability to cause disease but that the capacity for virulence may be a general feature of Caulobacters. All previous studies looking at the *in vivo* pathogenic potential of *Caulobacter* used murine cell lines or immunocompetent mouse models [20, 37]. In contrast, all clinical reports of human *Caulobacter* infections occurred in hospital settings with patients who are likely immunocompromised [7–10]. Since different bacteria may be virulent in different contexts, using a variety of host models may prove beneficial for understanding the contexts in which a bacterium can be toxic. For example, characterizing pathogenesis in *Galleria* enabled us to identify LPS as a causative agent of *C*. *crescentus* and *C*. *mirare* toxicity (Fig 4D). Since *E*. *coli* MG1655 also has LPS but is non-toxic, our studies further suggest that the relatively subtle chemical differences between largely conserved bacterial components can have significant implications for host interactions. In the future it will thus be important to directly determine if *Caulobacter* strains can cause disease in mammalian hosts in contexts that mimic clinically-relevant conditions such as immuno-suppressed states.

If *Caulobacter* species can be virulent towards *Galleria* and potentially even humans, why is the isolation of Caulobacters as human pathogens so rare? Typically, successful pathogens need to survive in the environment of their hosts [41]. *Caulobacter* is often described as an oligotroph since it is found in nutrient-poor environments such as fresh-water lakes and drinking water [4, 42]. However, our work shows that *Caulobacter* can also thrive in nutrient-rich culturing conditions (Fig 2). Metabolomic studies of the fluids from common infection sites such as peritoneal fluid, cerebral spinal fluid, and plasma show that these fluids contain metabolites and salt concentrations similar to those in media that support *Caulobacter* growth [43–45]. Thus, it is possible that *Caulobacter* species can survive in human hosts and that the reason they are not often detected is that they are not readily culturable on the media commonly used for clinical microbiology [46, 47]. Consistent with this hypothesis, we showed that *C*. *mirare* can be cultured on TSA blood agar while *C*. *crescentus* cannot, likely due to the increased salt tolerance of *C*. *mirare* (Fig 2). Moving forward, culture-independent identification methods such as mass spectrometry and metagenomic sequencing will help determine the true frequency of *Caulobacter* infections in humans [48, 49].

The ability of a classically-defined “non-pathogen” like *C*. *crescentus* to cause disease in the *Galleria* animal model and other Caulobacter species in hospital-acquired infections raises the question of what defines a pathogen. Can *any* bacterial species be considered pathogenic if given the right environment? Combining our findings with previous work on *C*. *crescentus* suggests that *Caulobacter* can carry out many of the processes typical of other pathogens, including biofilm formation, antibiotic resistance, killing of non-self bacteria, and *Galleria* host killing [7, 10, 50, 51]. Unlike the patient-isolated *C*. *mirare*, the CB15 *C*. *crescentus* strain studied here is an environmental isolate from a freshwater lake [2]. The ability of this environmental isolate to retain pathogenesis towards an animal host suggests that Caulobacters can survive in multiple niches [3, 41]. Both *C*. *crescentus* and *C*. *segnis* lack obvious host invasion factors, suggesting that their pathogenesis requires a compromised host and explaining why they are opportunistic pathogens. Furthermore, the killing of *Galleria* by *C*. *crescentus* does not require these bacteria to grow in the host, suggesting that its mere presence in certain environments is sufficient for pathogenesis. A recent opinion article suggested that pathogenesis should be viewed as a spectrum and that most bacteria will be pathogenic if present at a sufficient concentration to be deleterious to a host [52]. Our study supports this perspective, suggesting that broadening how we identify and isolate pathogens in clinical settings will allow us to better understand the spectrum of pathogens that actually infect humans. Elucidating the pathogenic potential of more bacteria and the mechanisms by which they cause disease will thus ultimately help combat infection as it arises in many contexts.

## Materials and methods

### Bacterial strains and growth conditions

For this study, an overnight culture is defined as a single colony inoculated in 5 ml tubes and grown for 16 hours. Exponential phase cultures were obtained by a 20-fold back dilution of overnight culture in fresh media and grown to an OD_660_ of ~0.5. *Caulobacter crescentus* laboratory strains (CB15 and NA1000) were grown in shaking culture at 30°C in PYE media on platform shakers. *Caulobacter mirare* (SSI4214) and *Agrobacterium tumefaciens* (A136) was grown in nutrient broth (NB) or LB medium, respectively, at 30°C in shaking culture. *E*. *coli* (MG1655 and MC4100 *wbbL+*) and *Pseudomonas aeruginosa* (PA14) were grown at 37°C in LB medium either in shaking culture or roller drum, respectively. Components of organisms’ respective growth media as well as other medias for agar plating have been described previously [53–54].

### Genomic analysis and phylogeny construction

Paired-end 150 nt Illumina MiSeq sequencing was performed on all samples at Princeton University’s Genomics Core. Scaffolds were generated from reads using UniCycler default settings on “normal mode,” and assembly metrics were compiled using QUAST [55]. Annotation of the genome was accomplished via DFast with default settings [21]. For phylogenetic construction, an online pipeline (www.phylogeny.fr) was used with default settings. Alignment via MUSCLE was run on “full mode” and phylogeny was determined by bootstrapping with 100 runs. Visualization of the tree was created using TreeDyn [56].

### *Galleria mellonella* healthspan assay

All *Galleria mellonella* larvae were Vita-Bugs© distributed through PetCo© (San Diego, CA) and kept in a 20°C chamber. Larvae were used for healthspan assays within three days of receipt of package. Worms which were not already melanized were assigned randomly to infection or control cohorts. All inoculums were administered using a sterile 1 ml syringe attached to a KD Scientific pump. Same volume injections (5 μL) were delivered at a rate of 250 μl/min to the fourth leg of the worm, which was sterilized with ethanol. Melanization phenotype was determined by observation of a solid black line along the dorsal midline of the larva (Fig 3A). Each figure graph is a representative cohort (n = 10–15 per treatment) from a biological triplicate, except for PA14 which was performed separately (Fig 4C). Mantel-cox statistics for the cohort were calculated using PRISM, and the pooled results are presented in the supplement (S3 Fig). For heat-killing experiments, exponentially growing bacteria were held at 100°C for 10 minutes. For serial dilution experiments, overnight cultures were diluted 4-fold in their respective medium. CFUs were determined by plating overnight cultures on agar plates. For conditioned media experiments, overnight cultures were centrifuged at 5700xg for 3 minutes and the resulting supernatant was injected into the worms. For LPS experiments, purified LPS was obtained as described previously and the final reaction mixture was injected into *Galleria* [57].

## Supporting information

S1 Fig Average Nucleotide Identity (ANI) plot between *Caulobacter* species. Histogram represents reciprocal best hits (two-way ANI) between fragments of the specified genomes with box-and-whisker plot showing the distribution.
(DOCX)

S2 Fig *ΔrsaA* healthspan. Galleria were injected with exponential growing (OD_660_ ~ 0.5) wild-type or S-layer deletion (*ΔrsaA* NA1000*)* mutants. Survival curve is a representative cohort (n = 12) of the experiment performed in biological triplicate. Pooled cohort data given with error bars representing standard error.
(DOCX)

S3 Fig Pooled cohort data for healthspan assay. Each experiment was performed in biological triplicate. n represents number of animals per cohort and error bars represents standard error.
(DOCX)
