# Supplementary material for: Both clinical and environmental Caulobacter species are virulent in the Galleria mellonella infection model
Source: PLoS One. 2020 Mar 12;15(3):e0230006. doi: 10.1371/journal.pone.0230006 (PMC7067423; doi:10.1371/journal.pone.0230006)
Supplement: S1 Fig — Histogram represents reciprocal best hits (two-way ANI) between fragments of the specified genomes with box-and-whisker plot showing the distribution. (DOCX) [file pone.0230006.s001.docx]

**Supplemental Figure 1:** **Average Nucleotide Identity (ANI) plot between *Caulobacter* species.** Histogram represents reciprocal best hits (two-way ANI) between fragments of the specified genomes with box-and-whisker plot showing the distribution.


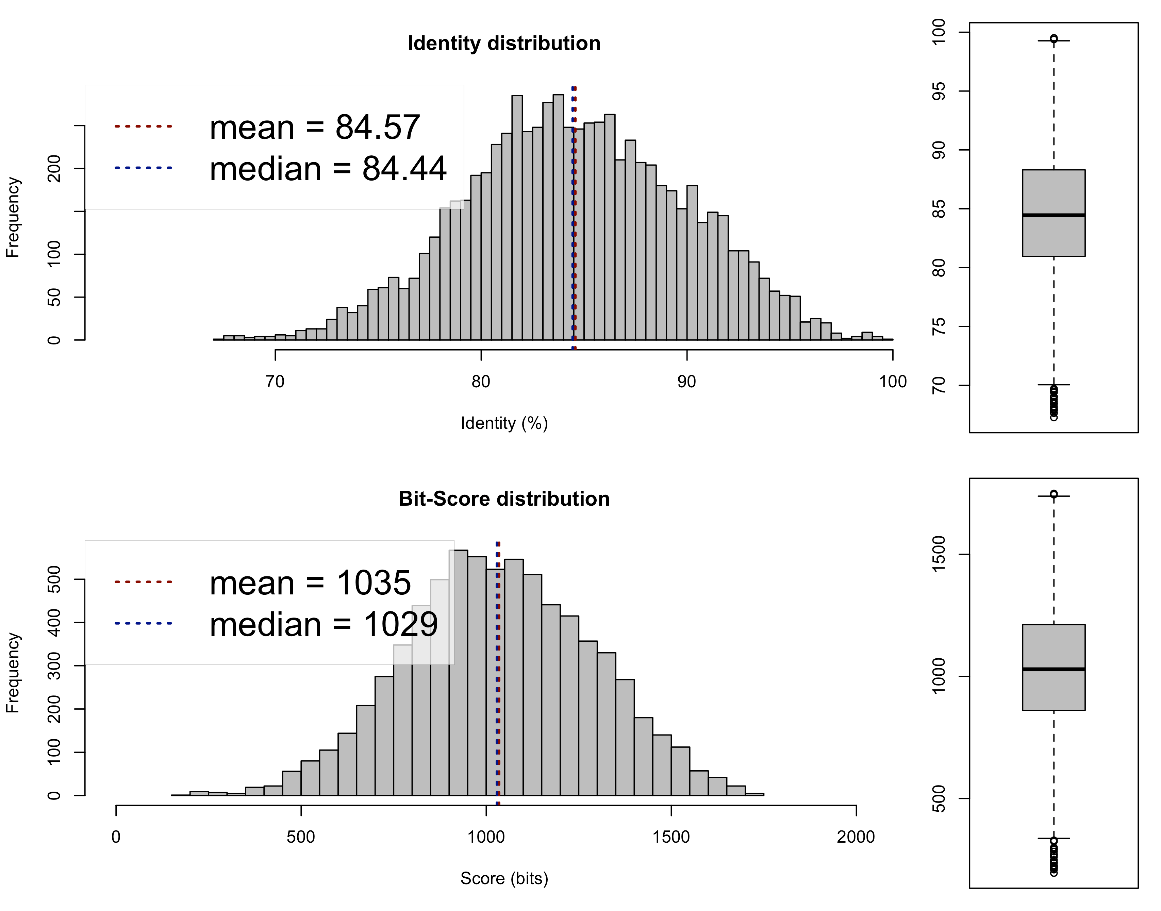

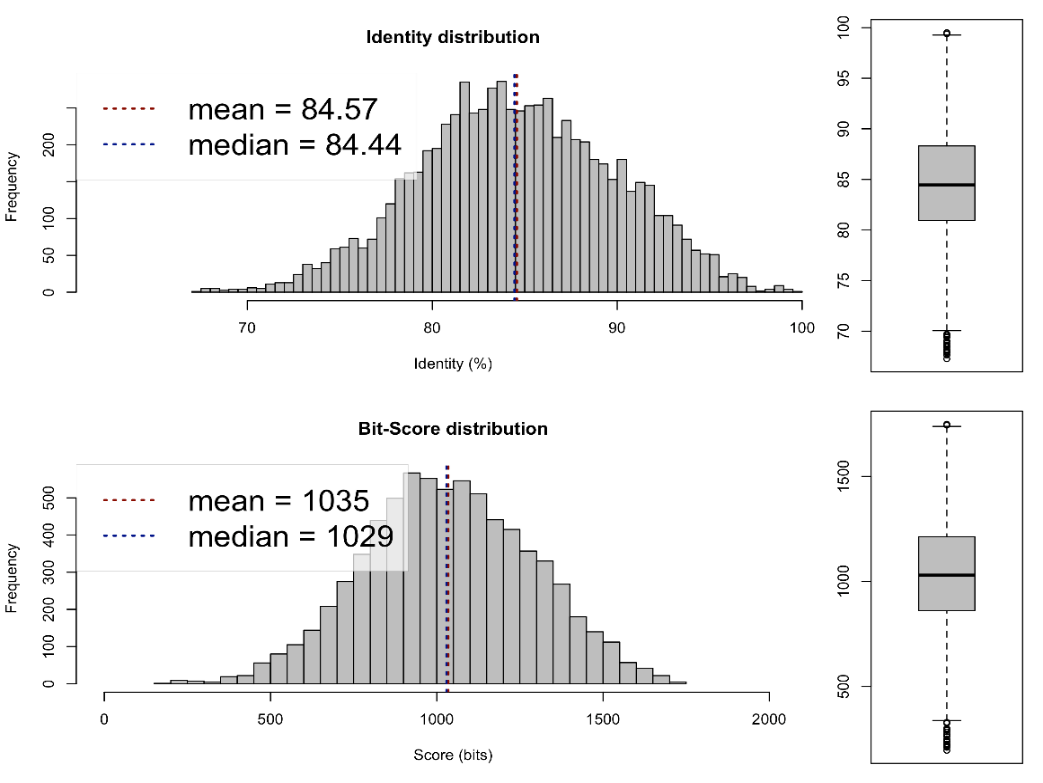


*Caulobacter segnis* ATCC21756 and *Caulobacter mirare* SSI4214


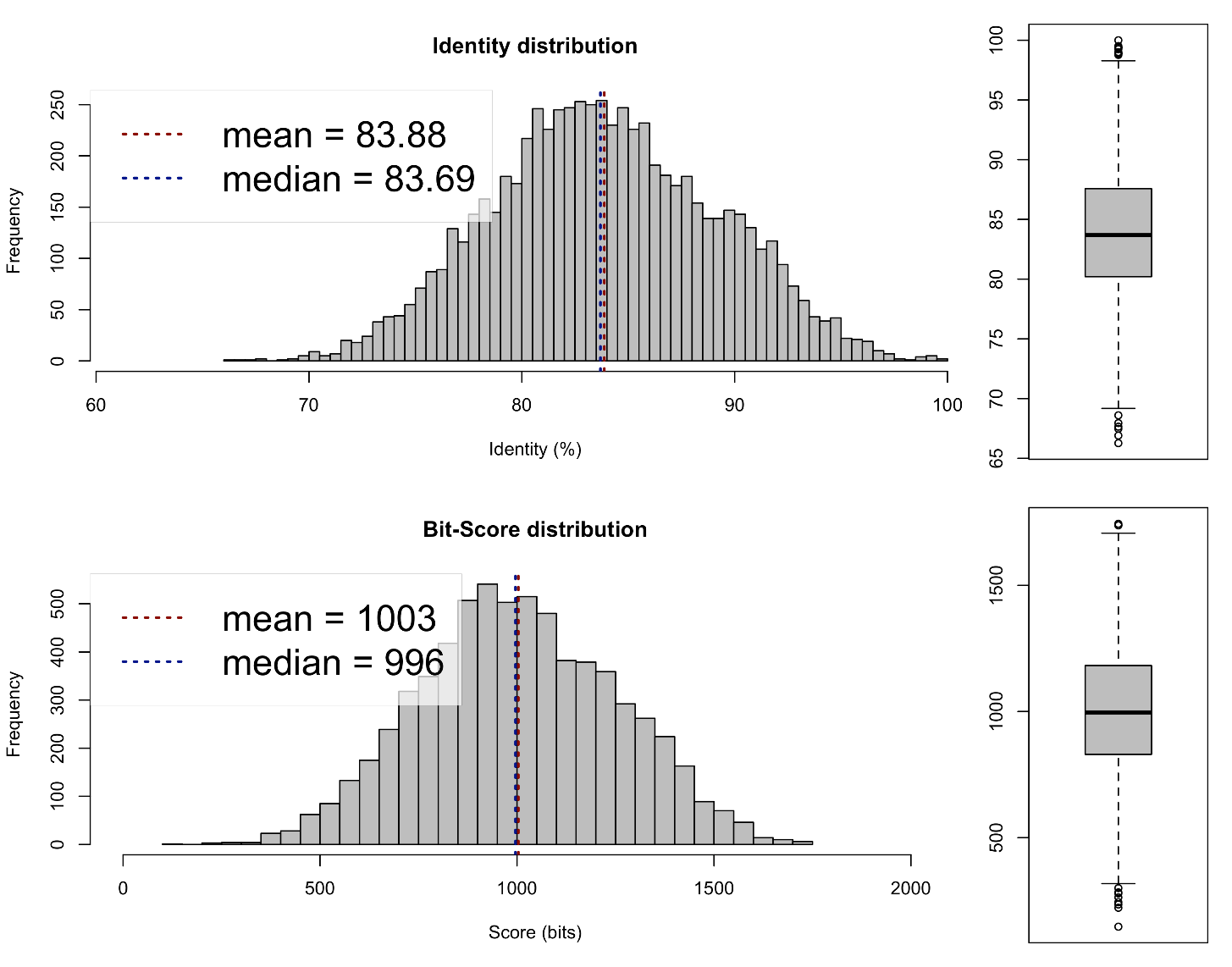

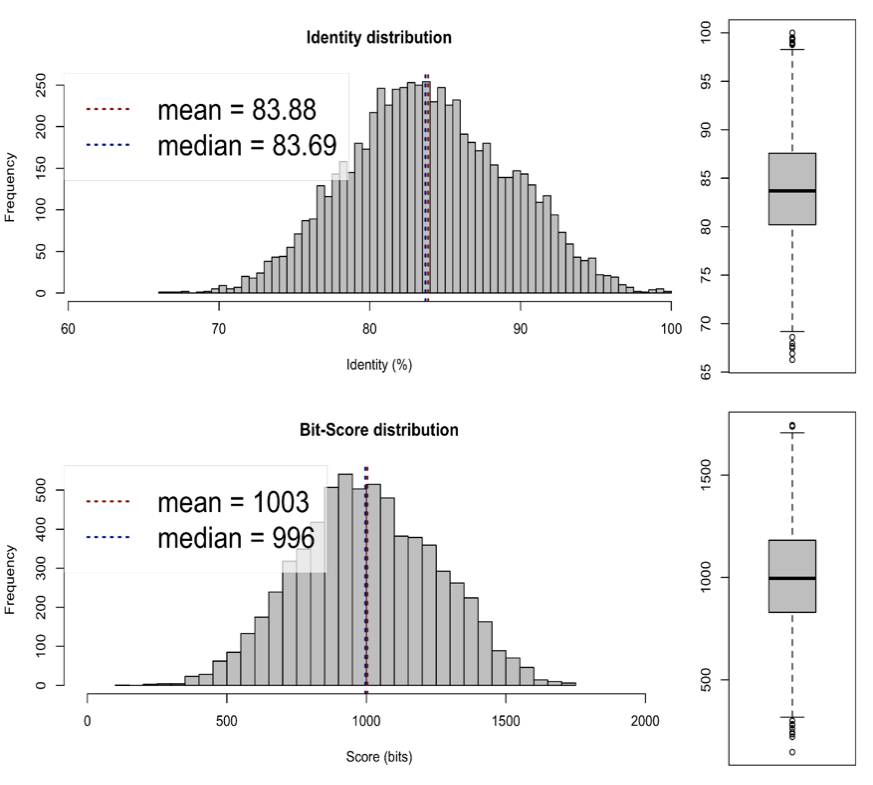


*Caulobacter crescentus* CB15 and *Caulobacter mirare* SSI4214


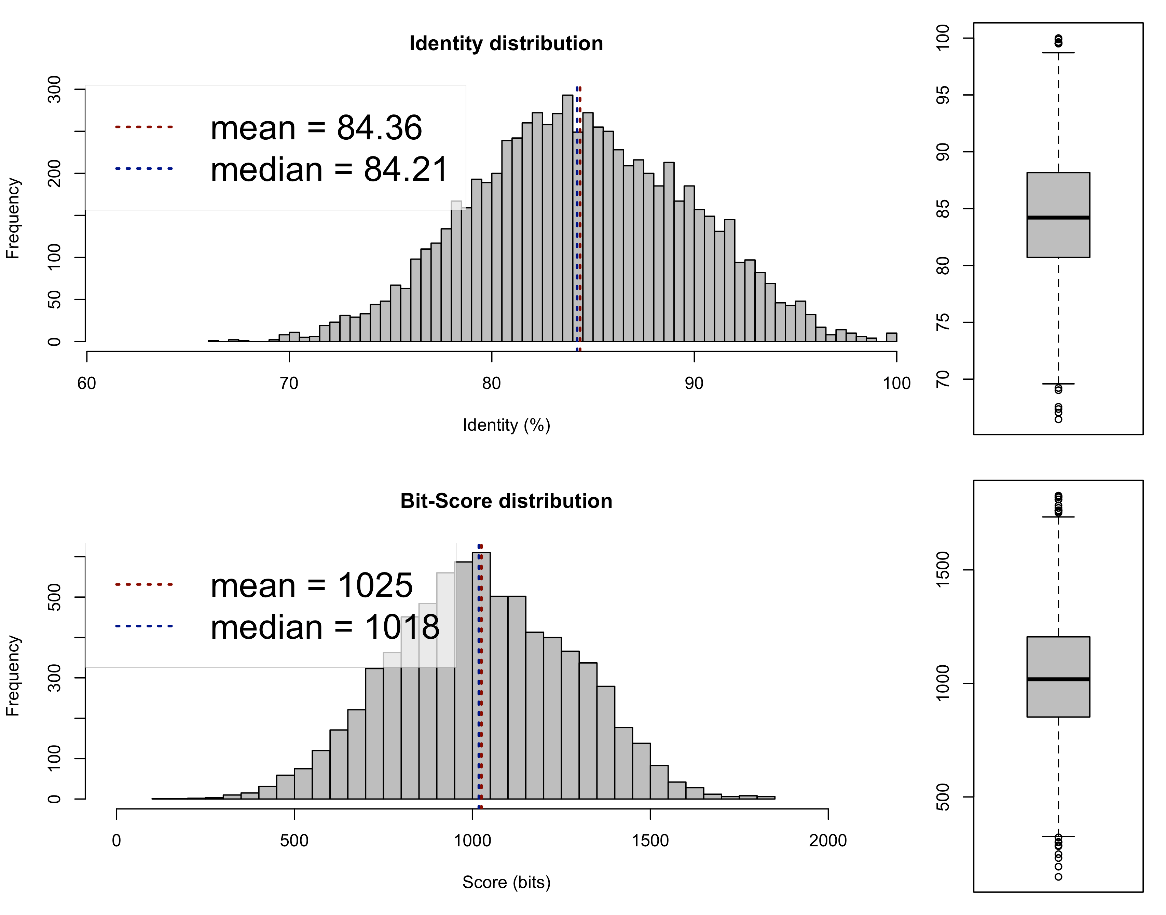

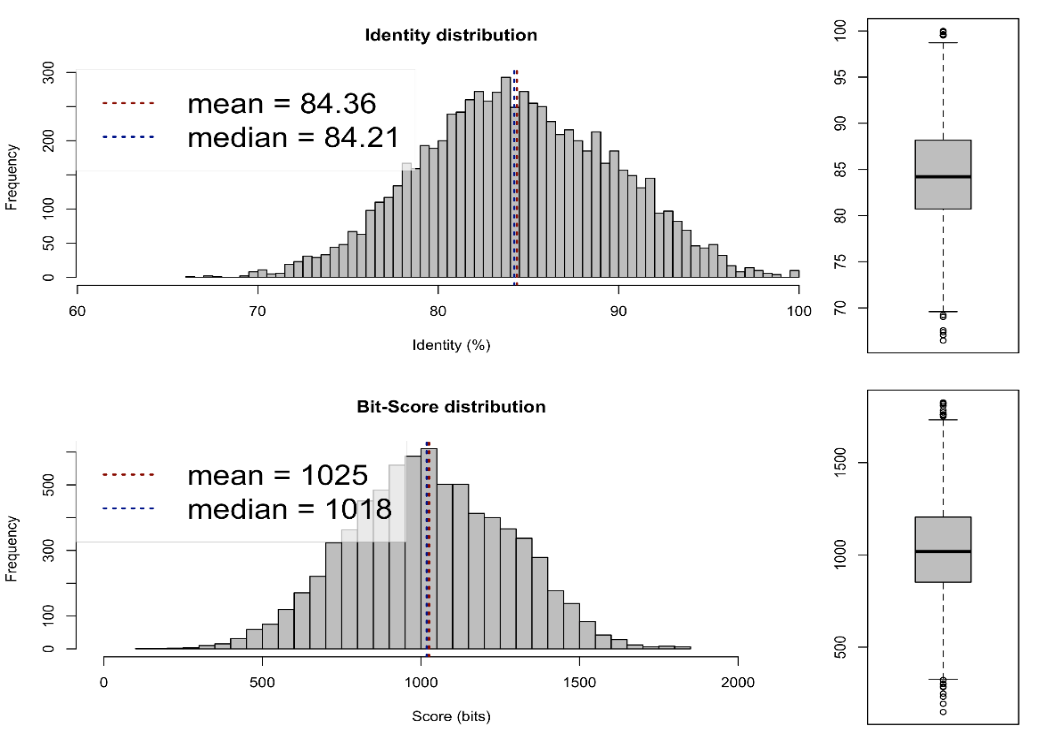


*Caulobacter crescentus* CB15 and *Caulobacter segnis* ATCC21756
