# Supplementary material for: Both clinical and environmental Caulobacter species are virulent in the Galleria mellonella infection model
Source: PLoS One. 2020 Mar 12;15(3):e0230006. doi: 10.1371/journal.pone.0230006 (PMC7067423; doi:10.1371/journal.pone.0230006)
Supplement: S2 Fig — Galleria were injected with exponential growing (OD660 ~ 0.5) wild-type or S-layer deletion (ΔrsaA NA1000) mutants. Survival curve is a representative cohort (n = 12) of the experiment performed in biological triplicate. Pooled cohort data given with error bars representing standard error. (DOCX) [file pone.0230006.s002.docx]

**Supplemental Figure 2:** ***ΔrsaA* healthspan.** Galleria were injected with exponential growing (OD_660_ ~ 0.5) wild-type or S-layer deletion (*ΔrsaA* NA1000*)* mutants. Survival curve is a representative cohort (n = 12) of the experiment performed in biological triplicate. Pooled cohort data given with error bars representing standard error.
