# Supplementary material for: Both clinical and environmental Caulobacter species are virulent in the Galleria mellonella infection model
Source: PLoS One. 2020 Mar 12;15(3):e0230006. doi: 10.1371/journal.pone.0230006 (PMC7067423; doi:10.1371/journal.pone.0230006)
Supplement: S3 Fig — Each experiment was performed in biological triplicate. n represents number of animals per cohort and error bars represents standard error. (DOCX) [file pone.0230006.s003.docx]

**Supplemental Figure 3:** **Pooled cohort data for healthspan assay.** Each experiment was performed in biological triplicate. n represents number of animals per cohort and error bars represents standard error.

Figure 2C (n = 15)

Figure 3A (n = 10)

Figure 3B (n = 12)

Figure 3C (n = 12)

Figure 3D (n = 12)
